# Fecal 16S rRNA sequencing and metabolomics reveal abnormal metabolism activity in preterm infants with different gestational ages

**DOI:** 10.3389/fcimb.2025.1530653

**Published:** 2025-05-26

**Authors:** Ling Liu, Liang Guo, Jincheng Dai, Xiangsheng Cai, Benqing Wu

**Affiliations:** ^1^ Department of Pediatrics, Affiliated Hospital of Guangdong Medical University, Zhanjiang, China; ^2^ Department of Neonatology, Guangming District People’s Hospital, Shenzhen, China; ^3^ Department of Pediatrics, Guangming District People’s Hospital, Shenzhen, China; ^4^ Clinical Laboratory, Guangzhou Eleventh People’s Hospital, Guangzhou Carde and Talent Health Management Center, Guangzhou, China

**Keywords:** gut microbiota, preterm infants, intestinal metabolites, microbial diversity, pyrimidine metabolism

## Abstract

**Objective:**

This study aims to conduct a comprehensive analysis of the differences in gut microbiota and metabolomics in preterm infants stratified by gestational age.

**Methods:**

Fresh fecal samples were collected from neonates within the first 3 days after birth. The gut microbiota composition and the changes in specific taxa abundance were analyzed using 16S rRNA sequencing. Metabolomic profiling was performed using liquid chromatography-tandem mass spectrometry (LC-MS/MS). Participants were categorized into four groups based on gestational age at birth: PreA group (34–36 weeks), PreB group (32–33 weeks), PreC group (28–31 weeks), and control group (37–42 weeks). Metabolic pathways were identified through metabolomics analysis, referencing the Kyoto Encyclopedia of Genes and Genomes (KEGG) database.

**Results:**

Notably, Principal Coordinates Analysis (PCoA) showed clear separation among samples from all groups, with significant differences noted in the PreC group when compared with the other three. We found a strong association between *Escherichia-Shigella* and *Ureaplasma* genera with infants born before 32 weeks of gestation, suggesting a higher risk of opportunistic infections for preterm infants under this gestational threshold. As gestational age increases, *Megamonas* and *Prevotella* gradually emerged, while *Escherichia-Shigella* and *Ureaplasma* progressively diminished. KEGG enrichment analysis indicated that Pyrimidine metabolism was a differentially regulated pathway between the PreA group and the control group. Interestingly, the only major differential metabolic pathway between the PreB group and the control group was Arachidonic acid metabolism. The bubble diagram revealed significant enrichment of differential metabolites in Pyrimidine and beta-Alanine metabolism pathways when comparing the PreC group with the control group.

**Conclusion:**

Significant differences were observed in the fecal microbiome and metabolome between preterm and full-term infants, particularly in those born before 32 weeks of gestation. These findings suggested that the gut microbial system in preterm infants undergone progressive maturation, approaching a “healthy” state characteristic of full-term infants as gestational age increases.

## Introduction

In recent years, research on the human gut microbiome has become a significant priority (Toubon et al., 2022). The gut microbiota forms a symbiotic community within the human intestinal tract. It metabolizes lipids, proteins, and indigestible elements while producing short-chain fatty acids (Sender et al., 2016). With advancements in 16S ribosomal RNA (rRNA) gene analysis of fecal samples, researchers have identified gut dysbiosis in various diseases, including inflammatory bowel disease, pneumonia, mastitis, cancer, metabolic syndrome, and allergic disorders (Chen et al., 2021; Zhao et al., 2021). Gut dysbiosis compromises the intestinal mucosal barrier, increasing susceptibility to pathogens and the occurrence of infections (Wang et al., 2021).

Preterm infants are particularly vulnerable to gut dysbiosis, which is linked to conditions such as necrotizing enterocolitis (NEC), growth retardation, cognitive impairment, and cerebral injury (Toubon et al., 2022). The intestinal microbial composition in preterm neonates shows temporal dynamics and instability, differing significantly from that of adults or full-term infants. Their gastrointestinal colonization process is more susceptible to disturbances from external factors, including postnatal antibiotic use, mode of birth, and feeding practices (Wang et al., 2020). The gut microbiota in preterm infants shows reduced microbial diversity and abundance, predominantly featuring opportunistic pathogens such as Enterobacter, Enterococcus, Staphylococcus, Klebsiella pneumoniae, Escherichia coli, and Pseudomonas aeruginosa. Additionally, colonization by *Bifidobacterium* and Bacteroides is delayed compared to full-term neonates (Hill et al., 2017; Korpela et al., 2018; Guitor et al., 2022; Hiltunen et al., 2022).

The relationship between an abnormal gut microbiome and health outcomes in preterm infants has been confirmed. Recent studies highlighted the critical role of early-life gut microbiota in modulating immune development and long-term metabolic health, particularly in preterm infants at high risk of neurodevelopmental disorders (Richarte et al., 2021; Chang et al., 2024). Despite advancements, existing studies often lack stratification by gestational age (GA), limiting insights into developmental-stage-specific dysbiosis. While prior work has focused on broad taxonomic shifts, the functional implications of gestational age-dependent metabolic pathways remain poorly understood. To better understand the gut microbiome’s composition and function, we performed a comparative analysis of microbial composition and metabolites. We used 16S rRNA gene sequencing alongside liquid chromatography tandem mass spectrometry (LC–MS/MS) in a prospective cohort of preterm infants across various GA as well as term infants. Furthermore, we investigated potential dysbiosis related to the gut microbiota structure and metabolites among preterm infants with differing GA.

## Materials and methods

### General information

This study involved newborn infants admitted to the Shenzhen Hospital of the Chinese Academy of Sciences from June 2021 to January 2022. Researchers collected demographic data from medical records. They categorized participants into four groups based on gestational age (GA) at birth: PreA group (34–36 weeks), PreB group (32–33 weeks), PreC group (28–31 weeks), and control group (37–42 weeks). Inclusion criteria required that newborns be under three days old, have not received antibiotics or probiotics, and that their legal guardians signed informed consent forms. Exclusion criteria included pregnant mothers who used microecologics or antibiotics within one week prior to delivery, preterm infants who underwent surgeries, and those with congenital malformations, genetic metabolic disorders, or other congenital anomalies. Maternal complications during pregnancy (e.g., preeclampsia, gestational diabetes) were recorded and excluded if they required antibiotic or immunosuppressive therapy. Furthermore, the control group had to demonstrate good health without the use of antibiotics, probiotics, or any other medications. Control group infants were exclusively vaginally delivered to minimize confounding effects of cesarean section on initial microbiota colonization. Researchers obtained written informed consent from each participant’s guardians before enrollment, adhering to the approval granted by the Ethics Committee of the Shenzhen Hospital of the Chinese Academy of Sciences (approval number: LL-KT-21234).

### Collection of stool samples

Stool samples were collected from postpartum individuals within three days post-delivery. Nursing personnel collected approximately 0.5–1g of fecal material using sterile spatulas, transferred into pre-weighed 2mL cryotubes, and immediately stored at -80°C. Samples were transported on dry ice to Novogene for 16S rRNA gene sequencing and metabolomics analysis to ensure integrity.

### DNA extraction and Illumina sequencing

The CTAB method was employed for the extraction of total genomic DNA from the samples. Specific primers (515F-806R for 16S V4) with barcodes were utilized to amplify distinct regions of the genes (16S V3-V4). For PCR reactions, Phusion^®^ High-Fidelity PCR Master Mix (New England Biolabs) along with forward and reverse primers at a concentration of 2µM and approximately 10ng template DNA were used. Subsequently, TruSeq^®^ DNA PCR-Free Sample Preparation Kit (Illumina, USA) was employed following the manufacturer’s instructions to generate sequencing libraries with added index codes. The quality assessment of the library was performed using Qubit@2.0 Fluorometer (Thermo Scientific) and Agilent Bioanalyzer 2100 system. Finally, an Illumina NovaSeq platform was utilized to sequence the library resulting in paired-end reads of length 250 bp.

### Gut microbial analysis

The raw tags were subjected to specific filtering conditions in order to ensure the accuracy of subsequent analysis (Bokulich et al., 2013) utilizing the QIIME software (V1.9.1, http://qiime.org/scripts/split_libraries_fastq.html) (Caporaso et al., 2010). Quality control measures were implemented during this process. Subsequently, the tags were compared against the Silva database (https://www.arb-silva.de/) using usearch software (https://github.com/torognes/vsearch/) (Rognes et al., 2016) for identification and elimination of any chimera sequences (Haas et al., 2011). Sequence analysis was performed using Uparse software (Uparse v7.0.100, http://drive5.com/uparse/) (Edgar, 2013). The filtered sequences were then clustered into operational taxonomic units (OTUs) with a similarity threshold of ≥97%.A representative sequence was selected for each OTU and further annotated accordingly. Alpha diversity analyses were conducted based on this normalized dataset to assess species richness and evenness in a sample using abundance indices (Chao1 and ACE), as well as diversity indices (Shannon and Simpson).Beta diversity, measured by weighted and unweighted unifrac distances, was calculated using QIIME software (Version 1.9.1). Cluster analysis was performed via principal component analysis (PCA) and Principal Coordinate Analysis (PCoA).

### LC–MS/MS analysis

The Vanquish UHPLC system (ThermoFisher, Germany) was coupled with an Orbitrap Q ExactiveTM HF-X mass spectrometer (Thermo Fisher, Germany) for sample analysis at Novogene Co., Ltd (Beijing, China). A 100 mg sample was taken and placed in an EP tube, followed by the addition of 500µL of an 80% methanol aqueous solution. The mixture was vortexed and then allowed to sit in an ice bath for 5 minutes. Subsequently, it was centrifuged at 15,000g for 20 minutes at 4°C. A specific volume of the supernatant was diluted with mass spectrometry-grade water to achieve a methanol concentration of 53%. The solution was then centrifuged again at 15,000g for 20 minutes at 4°C, and the supernatant was collected for analysis via LC-MS. Hypesil Gold column (100×2.1 mm, 1.9μm) was utilized for the samples using a linear gradient over a duration of 17 minutes at a flow rate of 0.2 mL/min. In positive polarity mode, eluent A consisted of 0.1% formic acid in water and eluent B comprised methanol. For negative polarity mode, eluent A contained ammonium acetate at a concentration of 5 mM with pH adjusted to 9.0 and eluent B remained as methanol. The solvent gradient was set as follows: 2% B, 1.5 min; 2-85% B, 3 min; 100% B, 10 min;100-2% B, 10.1 min;2% B, 12 min.Q ExactiveTM HF mass spectrometer operated under positive/negative polarity mode with spray voltage of 3.2 kV, capillary temperature of 320°C, sheath gas flow rate of 40 arb and aux gas flow rate of 10 arb, Funnel RF level of 40, Aux gas heater temperature of 350°C.

The UHPLC-MS/MS generated mass spectrometry raw data underwent processing using Compound Discoverer 3.1 (CD3.1, ThermoFisher) to align peaks, select metabolites, and quantify them. Statistical analyses were performed using the statistical software R (version R-3.4.3), Python (version 2.7.6) and CentOS (release 6.6). When the data were not normally distributed, attempts were made to normalize them using the area normalization method. The identification of these metabolites was accomplished by referencing the KEGG database (https://www.genome.jp/kegg/pathway.html), HMDB database (https://hmdb.ca/metabolites), and LIPIDMaps database (http://www.lipidmaps.org/). MetaX was employed for conducting principal components analysis (PCA) and partial least squares discriminant analysis (PLS-DA) (Wen et al., 2017).

Differential metabolites were identified as those exhibiting VIP>1, P value< 0.05, and fold change≥2 or FC ≤ 0.5. Volcano plots were generated using ggplot2 in the R language based on the log2 (FoldChange) and -log10 (p-value) of these metabolites to identify metabolites of interest. Pearson’s method was used in Rlanguage to analyze the correlation between these differential metabolites. The KEGG database was utilized for investigating functions and metabolic pathways associated with these metabolites, while metabolic pathway enrichment analysis was conducted by assessing ratios x/n>y/N for pathway enrichment and considering a metabolic pathway statistically significant if its P value <0.05.

### Statistical analysis

The statistical analyses were conducted using SPSS 24.0 software (SPSS, Inc., Chicago, IL, USA) and R software (Version 2.15.3). Demographic characteristics and delivery data were compared between groups using Student’s t-test, Wilcoxon rank-sum test or Mann-Whitney U test. Differences in categorical variables were determined using the χ² or Fisher’s exact test. A principal components analysis was employed to compare samples among groups and identify statistically significant species at each classification level. The comparison of four groups for alpha diversity was assessed by the Wilcoxon rank-sum test. Unique taxa or functions that distinguished the groups as statistically significant biomarkers were identified through linear discriminant analysis effect size (LEfSE) with an LDA cutoff of 4. Associations between gut microbiome composition and metabolites were demonstrated using Spearman correlation coefficient. A significance level of P < 0.05 was deemed appropriate.

## Results

### Clinical features of the participants

A total of 48 subjects meeting the inclusion criteria were enrolled at Shenzhen Hospital of the Chinese Academy of Sciences between June 2021 and January 2022.Stool samples were collected from neonates with a median birth weight of 2.36 kg (range: 1.1-3.7 kg) and GA ranging from 28 to 41 weeks (**Supplementary Table 1**). **Supplementary Table 1** provides an overview of the baseline characteristics for each group. The mean GA of the PreA, PreB, PreC and control groups were 35.89, 33.00, 30.12 and 39.64 weeks, respectively. The 48 newborn infants were divided into the control group (n=15), the PreA group (n =13), the PreB group (n=11) and the PreC group (n=9).

### Microbial diversity analysis

**Figure 1:**
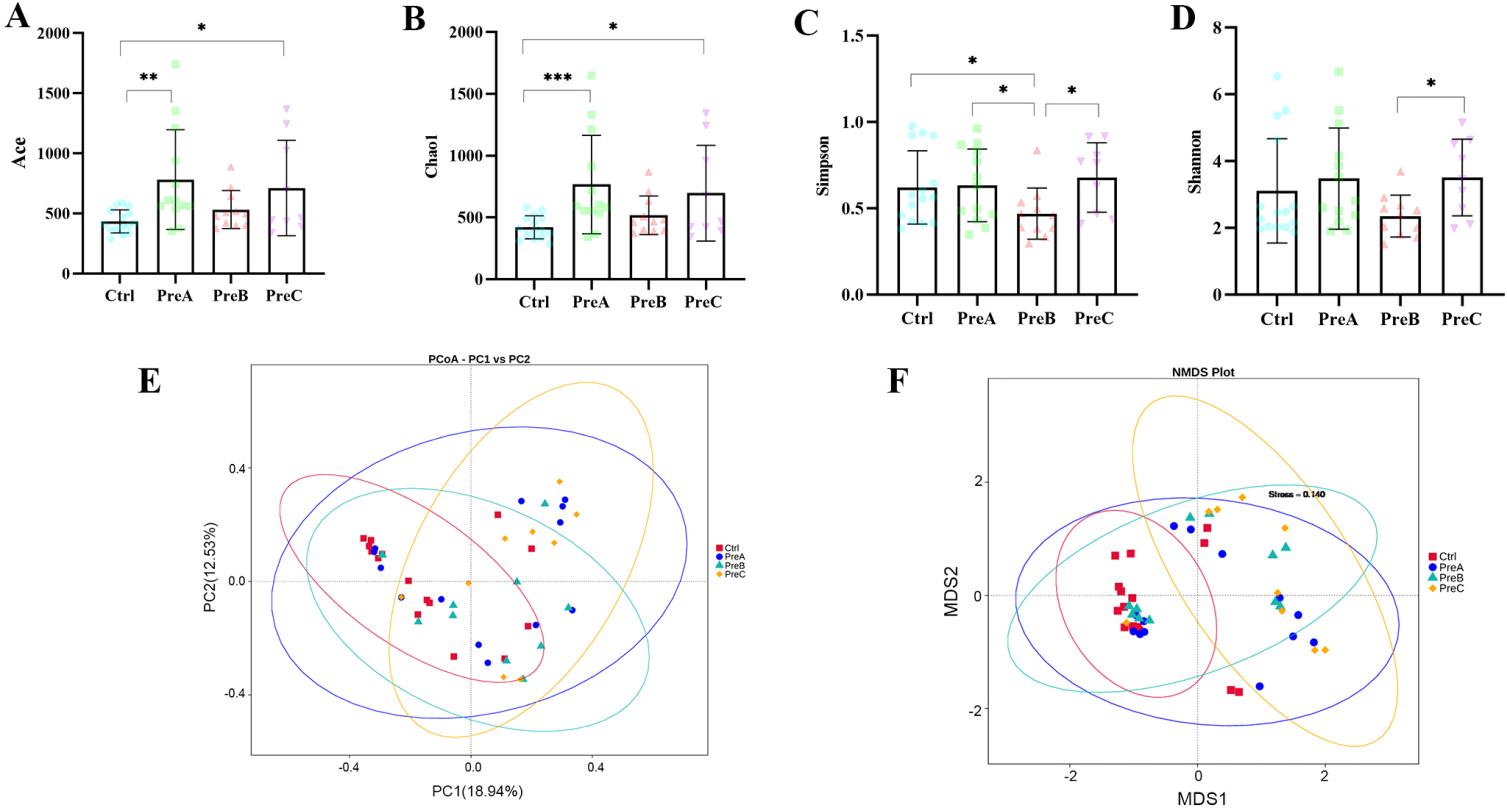
Alpha and beta diversity. ACE index **(A)**, Chao1 index **(B)**, Simpson index **(C)** and Shannon index **(D)**were used to assess alpha diversity. The Wilcoxon rank-sum test was conducted to compare these indices among the four groups. **(E)** PCoA analysis with the unweighted UniFrac distance of the microcosm composition. **(F)** Non-Metric Multi-Dimensional Scaling (NMDS) of the gut microbiota based on Bray–Curtis. *P<0.05, **P<0.01, ***P<0.001.

The alpha diversity, which quantifies the species richness and taxonomic diversity within a sample, was evaluated using multiple indices (ACE index, Chao index, Simpson index, and Shannon index).Our analysis revealed significant differences in ACE and Chao indices between the PreA group and control group (P=0.001 for **Figure 1A**; P=0.000 for **Figure 1B**), indicating that gut microbiota richness was higher in the PreA group than controls. Furthermore, the Wilcoxon rank-sum test demonstrated that both ACE and Chao indices were significantly elevated in the PreC group compared to controls (P=0.046 for **Figure 1A**; P=0.023 for **Figure 1B**). The Shannon index of PreB samples was lower than that of PreC samples (P=0.042 for **Figure 1D**), while the Simpson index of PreB samples was lower than all other groups (**Figure 1C**). Overall, our findings suggest a tendency towards greater alpha diversity of fecal microbiota in the PreA group relative to controls but no significant difference between the PreA and PreC groups based on these four indices.

The PCoA analysis revealed a distinct separation among the samples into four groups, with a significant distinction observed between the PreC group and the other three groups (**Figure 1E**). NMDS plots also indicated a significant difference in bacterial communities between the PreC group and the remaining groups (stress=0.14, **Figure 1F**). ANOSIM results demonstrated a significant dissimilarity in beta diversity between the PreB group and PreC group (R=0.118, P<0.05).Consistent with these findings, Amova analysis further confirmed an extremely significant variation in gut microbiota composition between the PreB/control group and PreC/control group (P<0.05). Our data suggested that as GA increases, preterm infants exhibit a gradual convergence of gut microbiota towards term infants. Notably, preterm infants born before 32 weeks displayed significantly different gut microbiota diversity compared to other gestational ages, which may contribute to subsequent digestive complications. Therefore, future research should prioritize investigating preterm infants born before 32 weeks of gestation.

### The comparative analysis of gut microbiota composition

**Figure 2:**
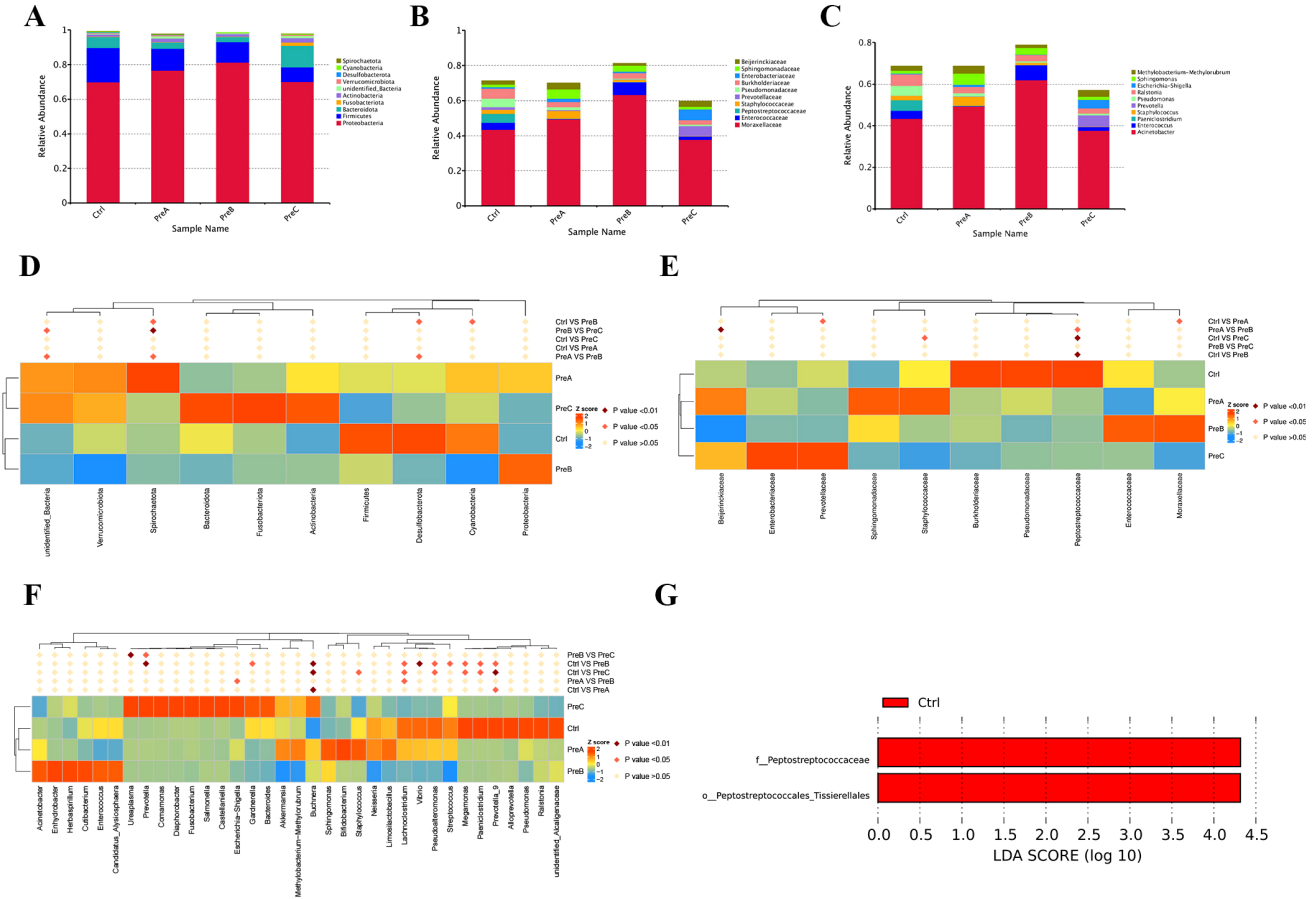
The distribution of gut microbiota was analyzed at different taxonomic levels including phylum **(A)**, family **(B)**, and genus **(C)**. Statistical significance between the groups was assessed using the MetaStat test for each taxonomic level- phylum **(D)**, family **(E)**, and genus **(F)**. **(G)** The distinct bacterial taxa across the four groups were illustrated using a Linear Discriminant Analysis (LDA) diagram, emphasizing only those species with an LDA>4 as significantly different. Red indicated the control group. LEfSe, linear discriminant analysis of effect size; LDA, linear discriminant analysis.

The most predominant phyla identified in the four groups were Proteobacteria and Firmicutes (**Figure 2A**). The control group exhibited the lowest relative abundance of Proteobacteria, while Firmicutes were found to be the most abundant. Additionally, it was observed that the PreC group tended to have a higher proportion of Bacteroidota and Actinobacteria, as well as a lower proportion of Firmicutes compared to the other three groups; however, no statistically significant differences were detected (P>0.05). Furthermore, an increase in Desulfobacterota and a decrease in Spirochaetota at the phylum level were observed in the control group when compared to the PreB group (P<0.05, **Figure 2D**).

At the family level, a significant decrease in Peptostreptococcaceae was observed in both the PreB and PreC groups compared to the control group (P<0.01, **Figure 2B** and **Figure 2E**). The analysis of flora composition at the family level revealed that Staphylococcaceae was more abundant in the PreA group than in the other three groups. Conversely, Moraxellaceae and Pseudomonadaceae showed decreased abundances compared to the other three groups; however, these differences were not statistically significant (**Figure 2E**). Notably, Enterococcaceae was exclusively present in the PreB group while Enterobacteriaceae and Prevotellaceae were found only in the PreC group. Burkholderiaceae and Pseudomonadaceae were enriched in the control group. As gestational age increases, Peptostreptococcaceae gradually emerged, while Prevotellaceae and Enterobacteriaceae progressively diminished, Moraxellaceae remained constant.

The most prevalent genera identified across all four groups were *Acinetobacter, Enterococcus, Staphylococcus* and *Paeniclostridium* (**Figure 2C**). In comparison to the other three groups, the control group demonstrated an elevation in *Paeniclostridium*, *Megamonas* and *Prevotella* at the genus level while *Buchnera* decreased (**Figure 2F**). Furthermore, a higher abundance of *Escherichia-Shigella* and *Ureaplasma* was observed in the PreC group (**Figure 2F**). The PreAgroup exhibited significantly increased relative abundances of *Bifidobacterium and Streptococcus* than the control group (P<0.05, **Figure 2F**). As gestational age increases, *Megamonas* and *Prevotella* gradually emerged, while *Escherichia-Shigella* and *Ureaplasma* progressively diminished.

The dominant floras were identified by analyzing all the samples using LEfSe (LDA>4). Compared to the other three groups, the control group showed a significant increase in the abundance of Peptostreptococcaceae (family) and Peptostreptococcales-Tissierellales (order) (**Figure 2G**). The LEfSe test indicated that Methylobacterium_brachiatum(species) had a significantly higher relative abundance in the PreA group compared to the control group (**Supplementary Figure 1**). Among the dominant fecal floras, it was observed that Peptostreptococcaceae (family), Peptostreptococcales-Tissierellales (order), and *Vibrio* (genus) were more prevalent in the control group than in PreB group (**Supplementary Figure 1**). Additionally, through LEfSe analysis, it was noted that Peptostreptococcaceae (family) showed greater abundance in the control group while Methylobacterium_brachiatum (species) displayed higher prevalence in the PreC group (**Supplementary Figure 1**).

### KEGG

**Figure 3:**
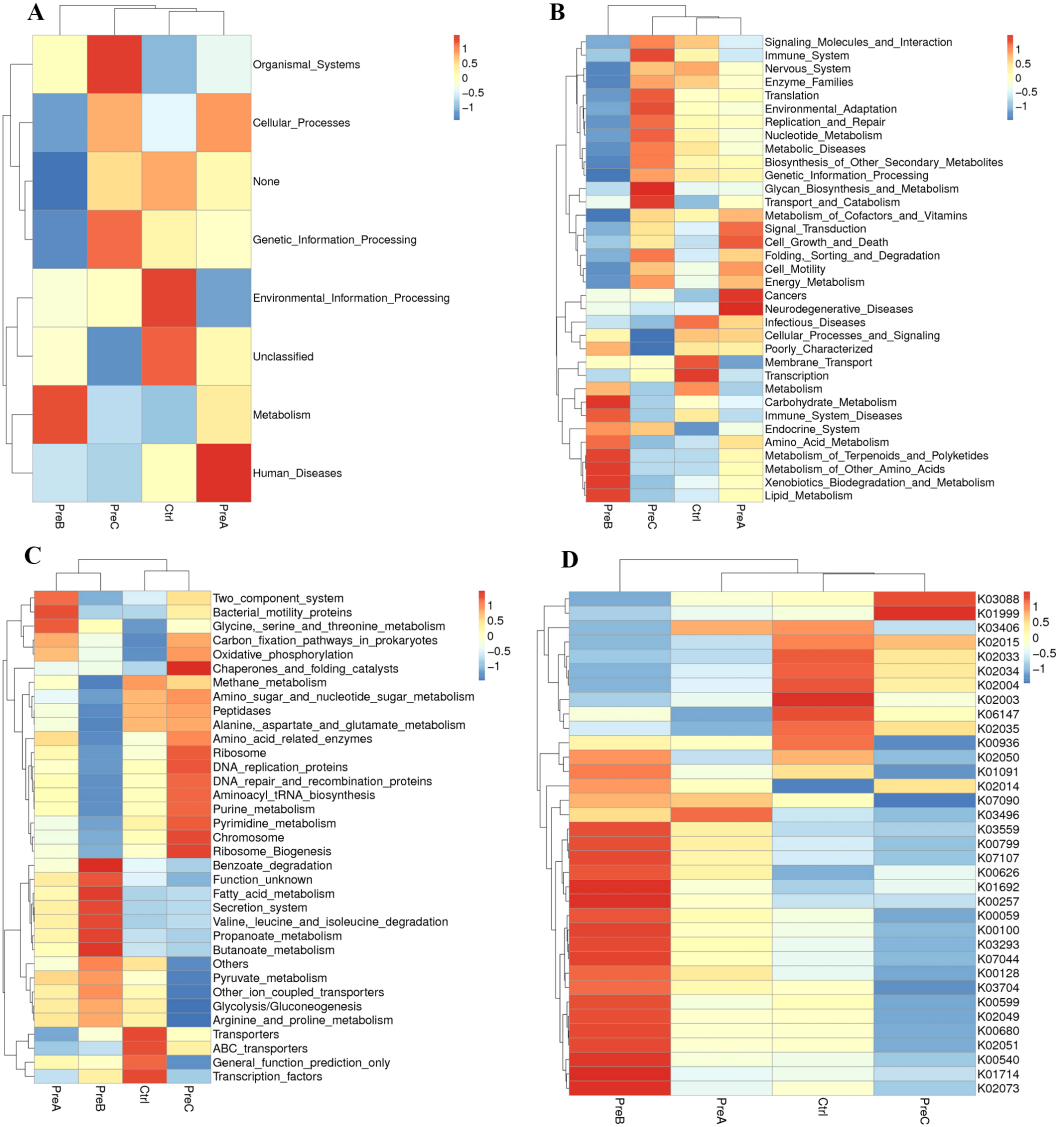
The average abundance of KEGG pathways differentially enriched in four groups according to level 1 **(A)**, level 2 **(B)**, level 3 **(C)** and KO **(D)**. Color gradient blocks were utilized to illustrate the variations in functional abundance across different groups. The closer the color was to red, the higher the abundance. KO: KEGG Orthology.

The heatmap analysis revealed disruptions in KEGG pathways at different levels (level 1, level 2, level 3, and KO) in preterm infants compared to the control group (**Figure 3**). The analysis of KEGG pathways indicated abnormalities in various aspects such as organismal systems energy, genetic information processing, cellular processes, metabolism, and human diseases in preterm infants. For instance, both organismal systems energy and genetic information processing were found to be higher in the PreC group than in the control group (**Figure 3A**). Notably, glycan biosynthesis and metabolism as well as transport and catabolism exhibited significant enrichment among the KEGG level 2 pathways of the PreC group; conversely, cancers and neurodegenerative diseases showed significant enrichment in the PreB group (**Figure 3B**). Our study demonstrated the enrichment for transporters, ABC-transporters, General_function_prediction_only and Transcription_factors within the control group’s KEGG level 3 pathways when compared to preterm infants (**Figure 3C**). Additionally, we observed a significantly higher abundance of unique genes such as RNA polymerase sigma-70 factor (K03088) and branched-chain amino acid transport system substrate-binding protein (K01999) in the PreC group compared to controls; however phosphoglycolate phosphatase (K01091) was lower in abundance with statistical significance (P<0.05, **Figure 3D**).

### The groups exhibit variations in their intestinal metabolites

**Figure 4:**
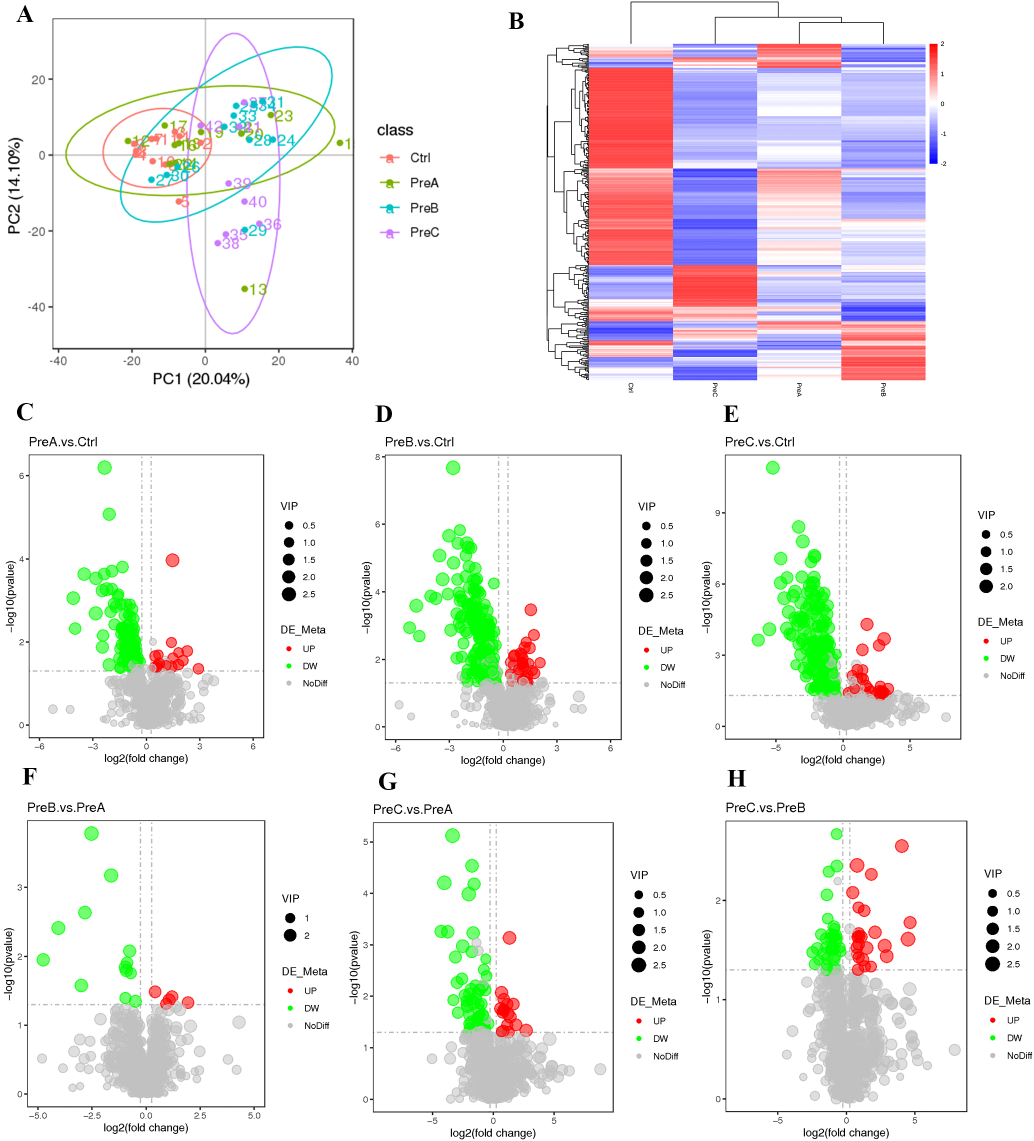
Changes in intestinal metabolites. **(A)** PCA of positive ion compounds. Each sample is represented by a point, and different groups are distinguished by various colors. **(B)** Cluster analysis of differential metabolites (positive ion compound). The expression levels of these metabolites are indicated by color, with blue representing low expression and red representing high expression. **(C–H)** a volcano map was created to visualize the differences in metabolites (positive ion compound): down-regulated differential metabolites are depicted in green, up-regulated ones in red, and those without any difference are labeled as gray.

The differential untargeted metabolomic substances in each group were analyzed using principal component analysis (PCA). Notably, a clear separation was observed between the PreC group and the other three groups, as illustrated in **Figure 4A**. The heatmaps exhibited distinct differences in intestinal metabolites among these groups (**Figure 4B**). Volcano maps revealed distinct screening results for positive ions, leading to the identification of 144 metabolites that exhibited differential abundance between the PreA group and control group. Among these metabolites, 18 were upregulated while 126 were downregulated in the PreA group compared to controls (**Figure 4C**). Specifically, a total of 229 differential metabolites were identified in the PreB group with 31 being upregulated and 198 downregulated relative to controls. Moreover, there were a total of 225 differential metabolites detected in the PreC group with 30 being upregulated and 195 downregulated when compared to controls (**Figure 4D**). The metabolites in PreB and PreC exhibited more pronounced disparities in their metabolic profiles as compared to term infants. The disparity in metabolite levels increases with the discrepancy in GA. Preterm infants displayed diverse degrees of metabolic downregulation compared to full-term infants.

**Figure 5:**
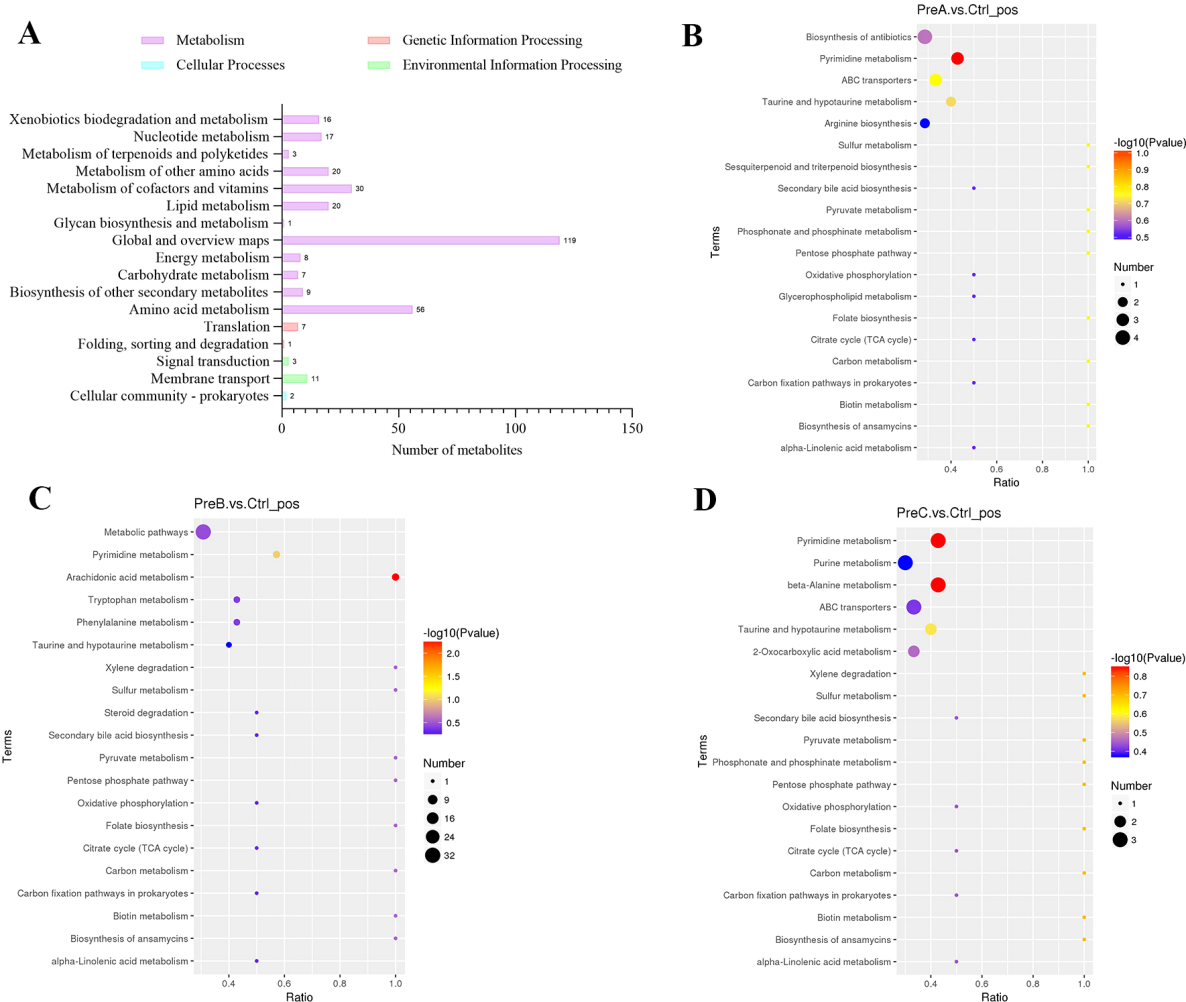
**(A)** Changes in metabolic pathways. KEGG pathway function annotation bar graph of positive ion compounds: the X-axis represents the number of metabolite annotations, while the Y-axis represents the annotated KEGG pathway. **(B–D)** Bubble plots for metabolic pathway enrichment analysis. The X-axis shows the enrichment factor (RichFactor), which is calculated by dividing the number of differential metabolites annotated to a specific pathway by the total identified metabolites annotated to that same pathway. A higher value indicates a greater proportion of differential metabolites being annotated to that particular pathway.

The identified differential metabolites were classified and annotated using the KEGG database to gain further insights into the functions performed by these specific metabolites. Our findings indicated that these differential metabolites were primarily involved in cellular processes, environmental information processing, metabolism, and genetic information processing (**Figure 5A**). Notably, significant differences were observed in the metabolite profiles between preterm infants groups compared to the control group (**Figure 5**). Through KEGG enrichment analysis, we discovered that pyrimidine metabolism was a distinct pathway associated with differential abundance of metabolites between PreA group and control group (**Figure 5B**). Similarly, only one main differential metabolic pathway was found between PreB group and control group-arachidonic acid metabolism (**Figure 5C**). The bubble diagram revealed significant enrichment of differential metabolites in pyrimidine metabolism and beta-alanine metabolism pathways when comparing PreC group to control group (**Figure 5D**). As gestational age increases, the metabolism of arachidonic acid progressively intensified, while pyrimidine metabolism gradually diminished, and the metabolism of secondary bile acids remained stable.

### The alterations in metabolic pathways are linked to the composition of gut microbiota

**Figure 6:**
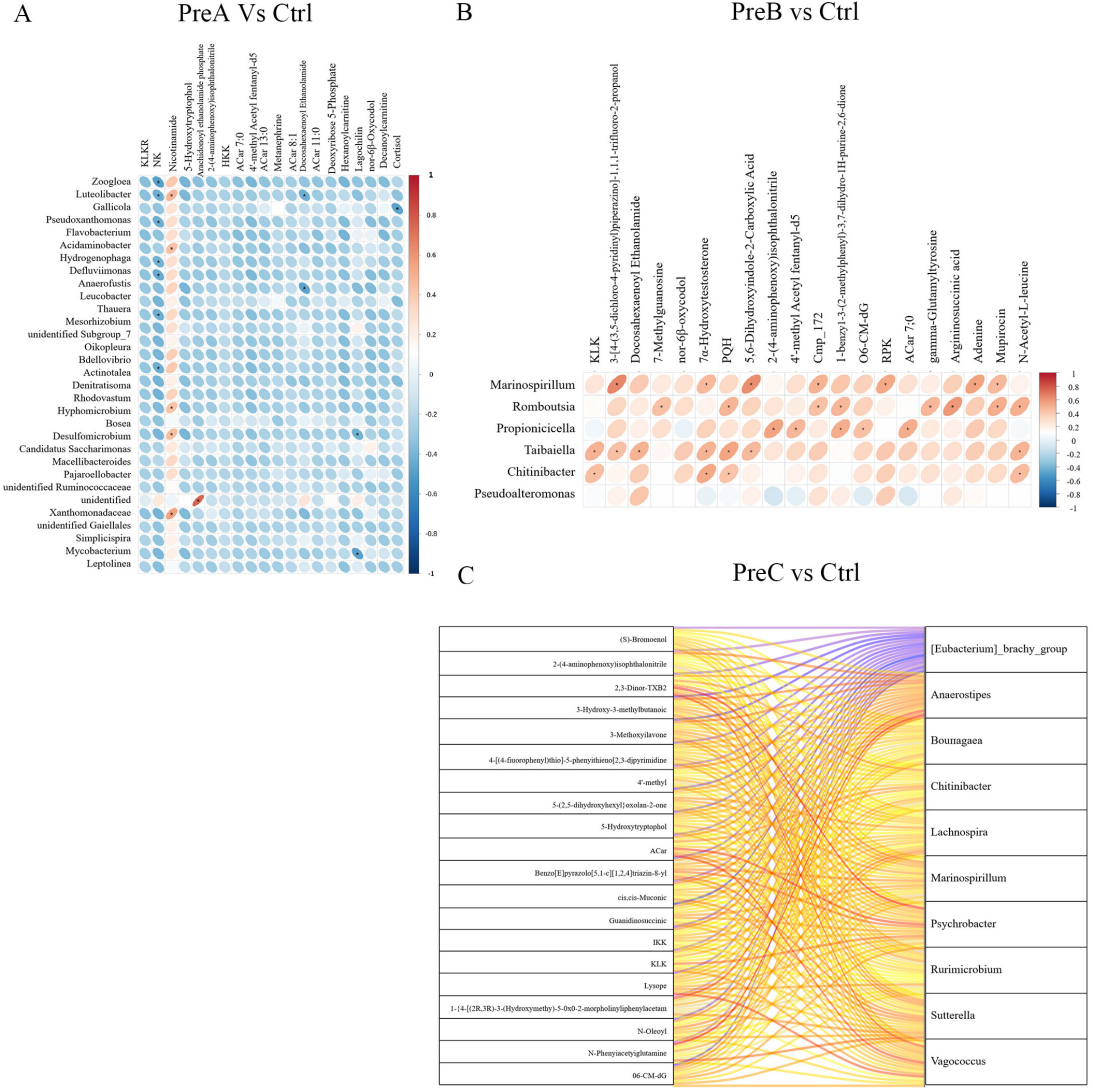
Correlation between gut microbiota and fecal metabolites of PreA vs control group **(A)**, PreB vs control group **(B)** and PreC vs control group **(C)**. Positive correlations are represented by red color, while negative correlations are indicated by blue color. The horizontal axis represents differential bacteria, while the vertical axis represents differential metabolites. The legend on the right is the correlation coefficient. A stronger positive correlation is depicted with a deeper shade of red, whereas a stronger negative correlation is illustrated with a darker shade of blue. The flatter the ellipse, the higher the absolute value of the correlation.

To investigate the relationship between fecal metabolites and gut microbiota in different groups, we performed a Spearman correlation analysis at the genus level. The results revealed significant correlations between *Zoogloea, Luteolibacter, Gallicola and Pseudoxanthomonas* with various metabolites in the PreA vs control group (**Figure 6A**). Moreover, *Marinospirillum, Romboutsia, Propionicicella, Taibaiella* and *Pseudoalteromonas* were found to exhibit significant correlations with different metabolites in the PreB vs control group. Furthermore, *Psychrobacter, Vagococcus, Anaerostipes, Marinospirillum* and *Lachnospira* demonstrated significant associations with distinct metabolites in the PreC vs control group (**Figure 6C**). There was a strong correlation (R=0.79) observed between *Anaerostipes* and N-Phenylacetylglutamine as depicted by **Figure 6C**.

The levels of control-enriched N-Phenylacetylglutamine, 3-Hydroxy-3-methylbutanoic acid, and cis,cis-Muconic acid were positively correlated with the abundance of *Agathobacter*, as depicted in **Figure 6C**.Furthermore, *Romboutsia* exhibited a positive association (R=0.585) with the level of control-enriched Argininosuccinic acid (**Figure 6B**). In **Figure 5A**, it can be observed that there is a positive correlation between *Acidaminobacter* abundance and control-enriched Nicotinamide levels (R=0.43). These findings suggested a close connection between the distinctive metabolites found in preterm infants and changes in gut microbiota composition. For instance, *Luteolibacter* displayed a positive correlation with Nicotinamide but demonstrated a negative correlation with RNK and Deoxyribose 5-Phosphate in **Figure 6A**.

## Discussion

To investigate the potential correlation between changes in gut microbiota and GAs, we conducted a comprehensive analysis of the gut microbiota and metabolomics in infants born with different GAs. As gestational age increases, *Megamonas* and *Prevotella* gradually emerged, while *Escherichia-Shigella* and *Ureaplasma* progressively diminished. Furthermore, we observed the metabolism of arachidonic acid progressively intensified, while pyrimidine metabolism gradually diminished as gestational age increases. This facilitates the progression of the gut microbiota system towards a state deemed “healthy” for full-term infants. These findings also underscored the importance of tailoring microbiota-associated treatments based on specific GAs.

The gut microbiota of preterm infants, characterized by reduced microbial diversity and increased colonization by pathogenic microorganisms, differed significantly from that of full-term infants (Westaway et al., 2022; Zhang et al., 2022). Previous studies had reported no significant difference in bacterial alpha diversities between moderately-late preterm infants and term infants (Chernikova et al., 2018). However, our study revealed substantial differences in the diversity of gut microbiota among the four groups. Additionally, PCoA analysis demonstrated a significant distinction between the PreC group and the other three groups. In our study, we observed an independent influence of GA on the microbiome of preterm infants. Preterm infants, especially those born before 32 weeks, faced challenges in establishing a healthy microbiome. These disparities were likely attributed to significant changes occurring in the gut microbiota of preterm infants during the initial 30 days after birth (Hui et al., 2021)as well as limited microbial diversity initially present in newborns which subsequently increased over time (Niu et al., 2020). The formation of neonatal gut microbiota begins at birth and continues to evolve throughout the first three years of life (Hui et al., 2021).

To further investigate the dominant strains within the four groups, we conducted a comprehensive analysis of the relative abundance of gut microbiota at various taxonomic levels in this study. In a healthy individual’s gut, Firmicutes and Bacteroides were primarily predominant, with smaller proportions consisting of Proteobacteria, Actinobacteria, Verrucobacteria, and Fusobacteria (Ho et al., 2020; Zhao et al., 2021). Previous research had demonstrated that Actinobacteria, Proteobacteria, Bacteroidetes, and Firmicutes were the predominant phyla found in preterm infants’ intestinal tracts (Zhang et al., 2022). Our findings suggested that the gut microbiome of newborns primarily consists of facultative anaerobes belonging to the phylum Proteobacteria, which was consistent with similar observations in infants from China (Niu et al., 2020). Although no statistically significant differences were observed, Proteobacteria abundance was relatively lower in the control group compared to other groups, whereas Firmicutes were more abundant. This implied that an increased presence of Proteobacteria may indicate dysbiosis and reflect an unstable structure within the intestinal microbial community (Shin et al., 2015). Previous studies had also demonstrated that during infancy, all infants tended to have a dominant population of Proteobacteria which gradually decreased until 24 months after birth (Yap et al., 2021). This transition can be explained by the gradual shift from a predominantly aerobic to an anaerobic gut environment in newborns, allowing for colonization by strict anaerobic bacteria such as *Bifidobacterium*, *Clostridium*, and *Bacteroides* (Matamoros et al., 2013; Arrieta et al., 2014).

Preterm infants born before 32 weeks of gestation exhibited the highest abundance of *Escherichia-Shigella* and *Ureaplasma*, with the most pronounced dysbiosis in their gut microbiota, alongside the highest levels of pyrimidine metabolism. The gut microbiota of preterm infants, particularly those born before 32 weeks of gestation, is highly susceptible to harmful bacterial infections and dysbiosis. *Megamonas* as a producer of short-chain fatty acids may reduce the abundance of opportunistic pathogens, such as *Escherichia coli* and *Shigell*, and may be beneficial for gut health, however, *Megamonas* may serve as a risk factor in specific diseases, such as colorectal cancer and neurodevelopmental disorders (Richarte et al., 2021). *Prevotella* in the gut can promote the production of short-chain fatty acids and can metabolize glycans present in certain foods and contribute to weight gain and improved intestinal function (Chang et al., 2024). *Escherichia-Shigella* are primarily harmful, causing a range of gastrointestinal illnesses and other health issues. *Ureaplasma* is generally considered a potentially harmful bacterium, and *Ureaplasma* infections during pregnancy may be associated with preterm labor and low birth weight (Olomu et al., 2009). At family level, Peptostreptococcaceae gradually emerged, while Prevotellaceae and Enterobacteriaceae progressively diminished. Previous studies had indicated that Lactobacillaceae and Peptostreptococcaceae played a crucial role in acetic acid production within rat intestines (Lu et al., 2022). It has been suggested that Peptostreptococcaceae acted as a beneficial microbe positively associated with acetic acid levels (Lu et al., 2022). Our findings supported probiotic supplementation (e.g., Bifidobacterium longum) tailored to gestational age, particularly for infants <32 weeks, to suppress opportunistic pathogens like *Escherichia-Shigella*.

Finally, we performed KEGG analysis to determine the functional annotations of genes and metabolic pathways. Significant alterations were observed in the pathways of arachidonic acid metabolism between the PreB and control groups, whereas pyrimidine metabolism was identified as a differential pathway between the PreA/PreC and control groups. The enrichment of arachidonic acid metabolism in PreB infants suggested potential targets for lipid-based nutritional interventions to mitigate inflammation. The gut microbiota activity modulated pyrimidine metabolism in preterm infants, which was closely associated with brain growth and metabolism (Pineiro-Ramos et al., 2021). Arachidonic acid metabolism has several beneficial effects on the human body, including its role in regulating renin release, modulating inflammation and immune responses, maintaining cardiovascular and renal health, and supporting neurological function (Gundala et al., 2017; Xu et al., 2020; Das, 2022). Pyrimidine metabolism is a critical biological process involved in the synthesis and degradation of pyrimidine nucleotides, which are essential components of DNA and RNA. Dysregulated pyrimidine metabolism is associated with several harmful effects on human health, including cancer, neurological disorders, hematological disorders, and immunological disorders (Mao et al., 2021; Zhang et al., 2024). Pyrimidine metabolism disruptions may impair DNA synthesis in rapidly developing tissues (e.g., brain), while arachidonic acid dysregulation could exacerbate inflammation in preterm infants. Targeting these pathways via probiotics (e.g., Bifidobacterium) or dietary interventions (e.g., omega-3 supplementation) warrants further investigation.

As gestational age increases, *Megamonas* and *Prevotella* gradually emerged, while *Escherichia-Shigella* and *Ureaplasma* progressively diminished. The gut microbiota of infants undergone significant changes as gestational age increases, with certain components remaining stable while others disappear, ultimately promoting the development of a “healthy” full-term infant. The stable presence of beneficial bacteria like *Bifidobacterium* and the reduction of potentially harmful bacteria like *Escherichia-Shigella* and *Ureaplasma* contributed to a more balanced gut microbiome, which was associated with improved immune system development and a lower risk of inflammatory and autoimmune disease. The use of probiotics and prebiotics, as well as optimizing feeding practices and antibiotic use, can help support the development of a robust and balanced gut microbiota, ultimately improving the health outcomes of preterm infants (Lai et al., 2024; Huang et al., 2025). Further research is needed to fully understand the long-term effects of these interventions and to develop personalized approaches for managing the gut microbiota in preterm infants. These findings suggested that gestational age-specific probiotics (e.g., *Megamonas* or *Prevotella* strains) could restore microbial balance in preterm infants. Additionally, monitoring metabolites like arachidonic acid may serve as biomarkers for early intervention. Clinical trials integrating microbiota-directed therapies with metabolomic profiling are needed to translate these insights into practice.

It is important to acknowledge the limitations of the study results. Firstly, it should be noted that this study was conducted at a single center with a limited number of cases and fecal samples, especially PreC group. Future multicenter studies with larger cohorts are warranted to validate these findings. In the present study, we only collected meconium specimens and did not perform continuous monitoring of gut microbiota dynamics. Thirdly, numerous studies have indicated variations in the composition of gut microbiota between preterm and term infants. To further investigate our findings, future research should consider experimental studies and longitudinal analyses.

## Conclusion

Our findings indicated that there were significant differences in the fecal microbiome and metabolome of preterm infants compared to full-term infants, particularly among those born before 32 weeks of gestation. It is therefore imperative to identify treatments that are tailored to the specific associations with microbiota for different gestational ages.

## Data Availability

The amplicon sequencing data are available in the NCBI Sequence Read Archive (SRA) database (BioProject: PRJNA1095214). The detail data and materials available please see https://www.ncbi.nlm.nih.gov/bioproject/PRJNA1095214.
