# Supplementary material for: Fecal 16S rRNA sequencing and metabolomics reveal abnormal metabolism activity in preterm infants with different gestational ages
Source: Front Cell Infect Microbiol. 2025 May 26;15:1530653. doi: 10.3389/fcimb.2025.1530653 (PMC12146292; doi:10.3389/fcimb.2025.1530653)
Supplement: Supplementary file 1 [file Table1.docx]

Table 1s Demographic characteristics for the participants

| Characteristic | PreA(n=13) | PreB(n=11) | PreC(n=9) | Control(n=15) | P |
| --- | --- | --- | --- | --- | --- |
| Gestational age(week) | 35.89*±*0.16 | 33.00*±*0.18 | 30.12*±*0.50 | 39.64*±*0.24 | 0.000* |
| Birthweight(kg) | 2.37*±*0.12 | 1.96*±*0.08 | 1.35*±*0.05 | 3.26*±*0.06 | 0.000* |
| Gender male, n (%) | 7 (53.8%) | 6 (54.5%) | 8(88.8%) | 7(46.6%) | 0.215 |

*P＜0.05, **P＜0.01
