# Supplementary figures and images for: Fecal 16S rRNA sequencing and metabolomics reveal abnormal metabolism activity in preterm infants with different gestational ages
Source: Front Cell Infect Microbiol. 2025 May 26;15:1530653. doi: 10.3389/fcimb.2025.1530653 (PMC12146292; doi:10.3389/fcimb.2025.1530653)

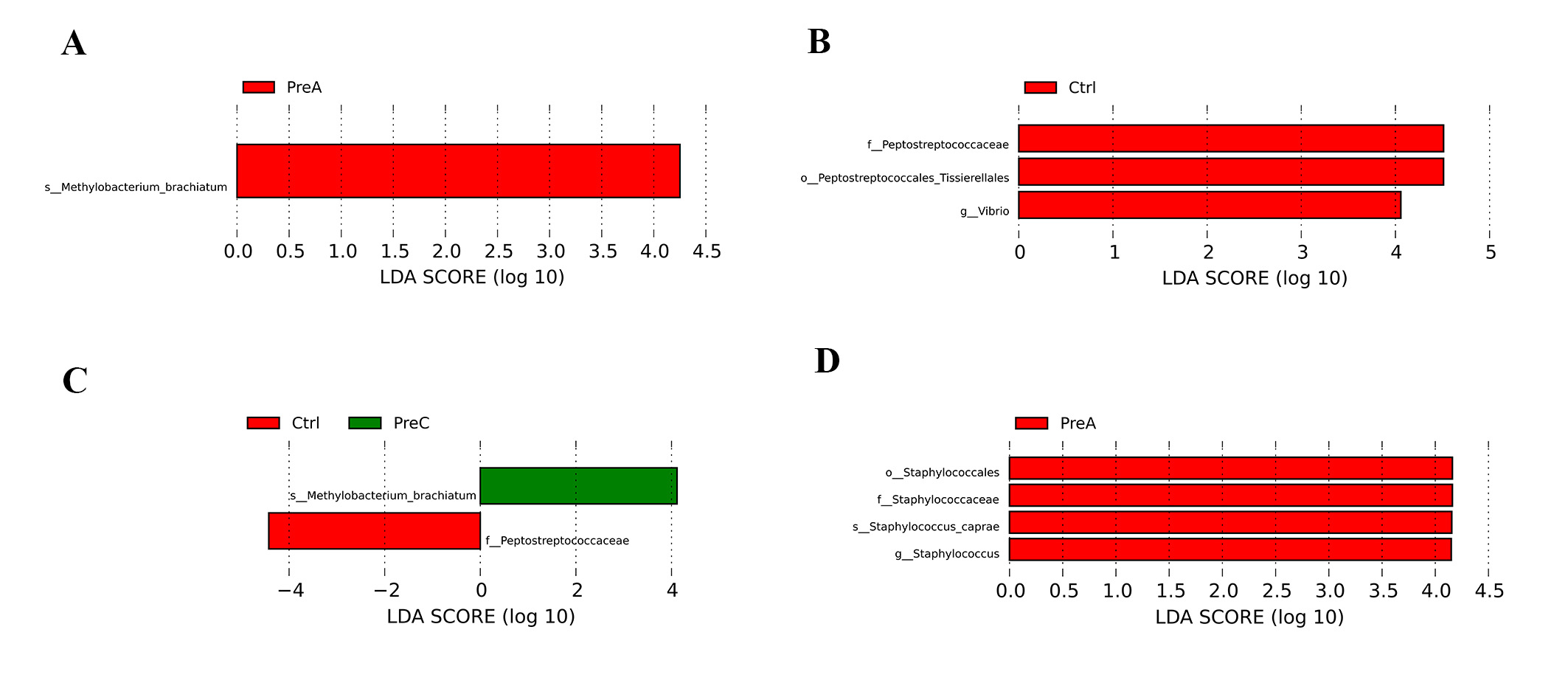

Supplement: Supplementary file 2 [file Image1.jpg]
